# Research priorities in the field of interprofessional healthcare education: a James Lind Alliance Priority Setting Partnership

**DOI:** 10.1136/bmjopen-2025-113426

**Published:** 2026-05-14

**Authors:** Christine Kumlien, Elliotte Ask, Malin Axelsson, Petri Gudmundsson, Olivia Hugosson, Jenny Jakobsson, Maria M Stollenwerk, Elisabeth Carlson

**Affiliations:** 1 Department of Care Science, Malmö Universitet, Malmö, Sweden; 2 Biomedical Science, Malmö Universitet, Malmö, Sweden

**Keywords:** Education, Medical, Health Services, Quality in health care, Health Workforce, Methods

## Abstract

**Abstract:**

**Objectives:**

Interprofessional education (IPE) is a key factor, preparing students for collaboration to improve quality in healthcare. Current literature implies that IPE research needs to be relevant for students, teachers and stakeholders ensuring that research answers the most important research issues. Therefore, the objective of this study was to establish outcomes of a partnership between students, teachers and clinicians to rank the top 10 research priorities for IPE.

**Design:**

James Lind Alliance Priority Setting Partnership (JLA).

**Setting:**

Higher health education in Sweden.

**Participants:**

Students, teachers and healthcare professionals (clinicians).

**Method:**

According to the JLA process, a steering committee was established. A pilot survey to gather research uncertainties highly relevant for participants was performed and tested by the content validity index. The pilot survey was followed by a main survey with 53 participants and a final workshop to determine the top 10 research priorities.

**Results:**

The content validity index was satisfactory for 23 out of 27 research uncertainties, followed by minor changes and removal of three uncertainties. After processing the 24 uncertainties from the main survey, 21 remained in the workshop. The final top 10 research priorities included measurements to evaluate IPE, promoting and hindering factors for IPE, educational models for IPE, longitudinal studies on effects from IPE and implementation of IPE.

**Conclusion:**

The priorities represent consensus areas from students, teachers and clinicians to guide future research and justify and inform strategic allocation of research funding.

**STRENGTHS AND LIMITATIONS OF THIS STUDY**

- This study followed a robust and established methodology and provided an actual list of research priorities to address in future research aimed at interprofessional education.
- The James Lind Alliance structure proved to be valuable for capturing perspectives from different groups and for identifying pedagogical research priorities.
- The lack of patients in the priority setting partnership could be a limitation, as patients are important team members, and their engagement in interprofessional clinical education is essential for healthcare students.
- The top 10 list of research priorities may to some extent reflect a Swedish context and therefore may not be entirely applicable to other countries.

## Introduction

 In recent decades, the gap between groups experiencing health or ill-health has increased both globally and locally. Therefore, creating conditions for equal health are important prerequisites for sustainable social development. Furthermore, a rapidly changing demographic distribution where people are getting older with increasing risks for multiorgan diseases will likely lead to a greater demand for healthcare staff. In response to these challenges, interprofessional collaboration is increasingly important to reach the quadruple aim: reducing costs; and improving population health, the patient experience, team well-being and productivity.^1 2^ Those who work together in healthcare professions commonly consider themselves as interprofessional teams, even though they are only a group of individuals working alongside one another.^3^ However, teamwork requires that healthcare professionals understand each other and have the skills to collaborate. These skills are arguably not intuitive or learnt at work, and the current training of health professionals has been criticised for insufficiently preparing students for interprofessional collaboration. Therefore, it is recommended that interprofessional education (IPE) is interpolated throughout the continuum of health professions education.^2 4^ According to WHO’s definition, IPE occurs when students from more than one healthcare profession learn from and with each other for the explicit purpose of developing interprofessional collaborative practice to improve the quality of care and services.^5^ As a reflection of this, the entire field encompassing IPE and interprofessional collaborative practice is usually defined as IPE and collaborative practice.^6 7^

Accordingly, educational activities must be designed to enhance the students’ ability to develop as competent, collaborative professionals who are ready to work in the complex and rapidly changing healthcare of today. Theoretically, Dennick^8^ describes collaborative learning as experiential and constructivist, stressing the importance of learning as actors engaging in interpersonal communication and feedback rather than merely as spectators. Furthermore, the learning methods should promote student activity and prepare students for professional work by allowing them to actively take part in learning processes and build knowledge through interaction and collaboration in connection with actual social and health challenges in society. Consequently, a variety of research-informed teaching strategies must be developed, implemented and scientifically evaluated to facilitate healthcare students’ development of collaborative knowledge and skills. While there has been a substantial increase in published IPE research in recent years, the need for high-quality cross-sectional and longitudinal research to inform this gap in knowledge remains.^4^ However, research about educational initiatives often overlooks the shared priorities of students, teachers and the healthcare sector, in which the students are expected to work as professional clinicians after graduation. This happens, for example, when researchers’ priorities differ from those of the target group, which poses a risk that important and relevant research becomes neglected. Misdirected research does not benefit students, teachers or professional healthcare workers, nor does it contribute to better and safer care for patients.^9^

The importance of involving patients, their family members and the public has been highlighted internationally, and a recent shift ensures that research funding efforts are influenced by the major stakeholders and service users.^10^ In recent years, the James Lind Alliance (JLA) methodology, which was established to support priority setting partnerships,^9^ is recognised as one way to involve users. The JLA methodology offers a systematic process of transparency that aims to ensure that research answers the questions that users, peers, relatives and stakeholders agree reflect the most important research issues. Members in priority setting partnership groups consist of representatives from the group of interest who, together as equals, define research uncertainties within a specific area. The JLA process includes steps to sort and prioritise the research uncertainties until a list of top 10 uncertainties is reached in consensus. Previously, the JLA methodology was mainly used to study research uncertainties in diagnose-related populations.^11 12^ However, the JLA methodology has seldom been used to study pedagogical research priorities, and in this case, research uncertainties concerning IPE. Yet, a previous study involving students setting research priorities inspired by the JLA approach was used to identify the top 10 uncertainties for sleep research raised by students in higher education.^13^

To further emphasise the importance of user involvement in setting IPE research priorities, Khalili *et al*^4^point to how research teams should strive for the inclusion of learners, service users, community members and civil society as partners (eg, as informants, data interpreters, knowledge translators) in interprofessional research. The benefits are that these groups in their capacity as consumers of healthcare bring expert knowledge and insight perspectives and thus secure research that is relevant to end consumers and stakeholders.^7^ Moreover, Khalili *et al*^14^ argue that research on IPE should shift focus from the program-specific or project-specific level to determine the impact of IPE and integrate the perspectives and expectations of patients, clients and caregivers related to IPE. Thereby, the present project was initiated to combine the strengths of different scientific disciplines represented by teachers, students and healthcare professionals to identify priorities for research questions focusing on IPE. Consequently, the aim was to establish outcomes of a priority setting partnership between students, teachers and clinicians to identify the top 10 research priorities for IPE.

## Methods

**Figure 1:**
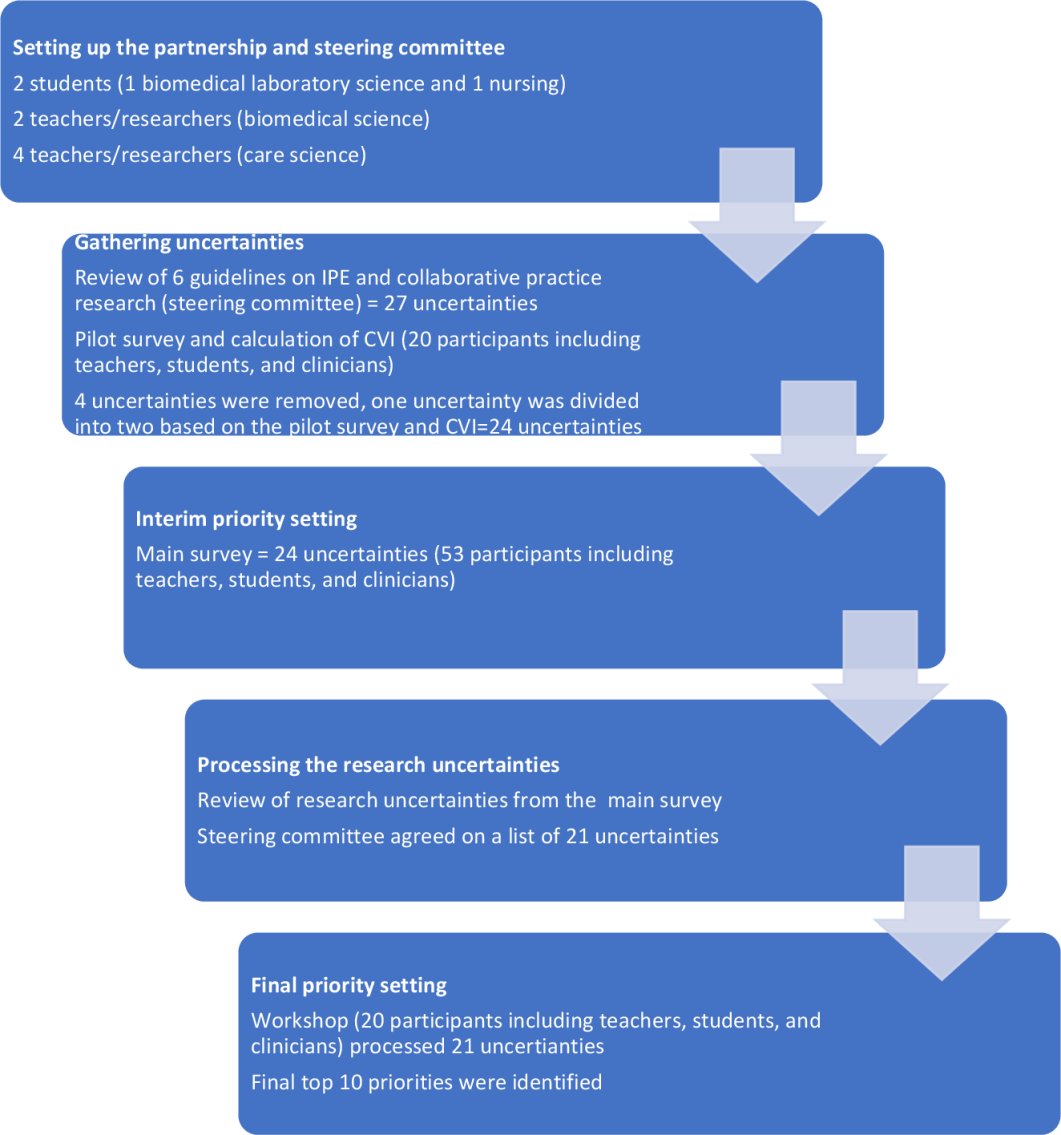
Overview of the priority-setting process for the top 10 research priorities for interprofessional education. CVI, content validity index; IPE, interprofessional education.

This is a project within the research platform; Research Informed Development of Higher education (RIDHE) (https://mau.se/en/research/research-platforms/ridhe/), which is an interdisciplinary platform for research on learning in higher education specifically targeting challenges in healthcare. The current project was initiated by the RIHDE steering group to identify the priorities for research questions regarding IPE and followed the JLA guidebook-recommended process (figure 1). The JLA method was considered as suitable for its inclusivity where those who do not usually raise their voice in research, in this case students, teachers and clinicians are included. Further reasons for using this methodology were the transparency of the process and the interaction part in the final priority setting workshop. Which in this case meant students and teachers who, together with clinicians, worked to identify and prioritise research uncertainties for IPE. The different steps in the process are described below.

### Setting up the partnership

A priority setting partnership was coordinated by the RIHDE steering group between students, teachers and researchers at the Departments of Care Science and Biomedical Science at Malmö University. For the partnership a steering committee was established, comprising eight persons: two students (one biomedical laboratory science and one nursing) who had applied as student representatives in RIHDE, four researchers/teachers (care science) and two researchers/teachers (biomedical science). The researchers were members of the RIHDE steering group. The committee was established in December 2022, when the aim and the JLA work process were presented. After the first meeting, a time plan was scheduled. The steering committee met four times during 2023 and communicated by email on a regular basis.

### Guidelines on IPE and collaborative practice research

The six most recent guidelines^414^,^18^ focusing on IPE and collaborative practice research were identified and reviewed by all members of the steering committee. According to the JLA guidebook, research uncertainties are typically informed by service users (students and teachers) with experience in the area of interest (IPE), healthcare professionals and existing guidelines and systematic reviews.^9^ Consequently, all identified research uncertainties, recommendations for future research and knowledge gaps extracted from the collected guidelines were discussed and refined by the steering committee. These, together with the committee members’ personal experiences as students and teachers, constituted the first 27 research uncertainties.

### Pilot survey

A digital pilot questionnaire with the 27 identified research uncertainties, four additional questions about age, gender, role (teacher, clinician or student), workplace and the possibility to suggest other important uncertainties was developed by the steering committee. Together with information about the importance of involving students, teachers and clinicians in prioritising research areas linked to IPE, the questionnaire was administered to a convenience sample of 35 people consisting of students, teachers and clinicians. Those were known and suggested by the members in the steering committee and were in the nearby environment. The respondents were further asked to evaluate importance on a 4-point Likert scale from 1 (not important at all) to 4 (very important) and whether it was easy to understand all aspects of the uncertainties from 1 (do not agree at all) to 4 (completely agree). A content validity index (CVI) was analysed according to item (I-CVI) and for the total scale (S-CVI). The I-CVI was computed for each item based on the respondent’s positive responses, the item rating score 3 or 4 on the Likert scale (thus dichotomising the ordinal scale into ‘relevant’ and ‘not relevant’), divided by the total number of respondents. The S-CVI average was then calculated by dividing the number of items with a positive response by the number of items. The recommended values for content validity index are I-CVI >0.78 for the item level and S-CVI >0.9 for the scale.^19^ Based on the pilot survey and CVI four uncertainties were removed, and one uncertainty was divided into two.

### Main survey

The main questionnaire consisted of 24 uncertainties about IPE with a 4-point Likert scale rated from 1 (not important at all) to 4 (very important) and the same additional questions as in the pilot survey regarding age, gender, role and workplace. It was also possible to add further uncertainties at the end of the questionnaire. The respondents could further register their interest in participating in the final workshop by adding their email address. Information about participation together with a link to the digital questionnaire was posted at the learning platform CANVAS online in September 2023 to students in the undergraduate nursing programme (semesters 4 and 5), the biomedical laboratory scientists’ programme (semester 4) and to clinicians taking part in an ongoing preceptor preparation course. The same information and the link to the questionnaire were also posted on the Faculty of Health and Society’s internal website for teachers. This recruitment strategy was estimated to reach approximately 600 possible participants. The digital questionnaire was developed and distributed in the digital tool, Microsoft Forms (Microsoft 365, Microsoft Corporation, Redmond, Washington). All data were anonymised and analysed descriptively, and exported to SPSS V.25 (IBM SPSS, Chicago, Illinois).

### Processing the research uncertainties

All the research uncertainties collected from the survey were processed and reviewed by the steering committee (figure 1). Unclear or out-of-scope uncertainties were removed at this stage. The remaining 21 uncertainties were included in the final workshop process.

### Final priority setting workshop

Students, teachers and clinicians who had registered their interest in participating in the final workshop were contacted by email. Written invitations with information about the aim of the workshop were sent out, emphasising the involvement in formulating future research questions regarding IPE. All persons who agreed to take part received an agenda and a list of the identified uncertainties before the workshop. Clear instructions were included to rank the uncertainties in advance and then discuss them during the workshop.

The workshop was conducted at Malmö University in a room specifically aimed for creative workshops. One of the steering committee members (CK) with previous experience from the JLA process chaired the workshop. The workshop started with a short presentation about the JLA process and the workshop. Thereafter, the participants were split into groups, with one facilitator in each group who led the discussions and took notes. In the first step, each participant shared their view on the uncertainties they felt most strongly and least strongly about. This was followed by a discussion where, together, the group members ranked the uncertainties.

In the second step, each group presented their rankings, which were then discussed by the whole group. The group reached agreement on the 15 most important uncertainties. In the third step, the participants were divided into the same groups where they discussed and decided the ranking for the 10 most prioritised research uncertainties. In the final step, the whole group compared and discussed the similarities and differences in their prioritising. A consensus approach was used when the participants were focused on agreeing on a top 10 list of priorities. The uncertainties were written on paper, with one uncertainty on each piece of paper, which made it possible for the participants to visually follow the mutual rankings of each uncertainty on the tables. The final rankings were visualised by all participants on the wall as the uncertainties shifted throughout the ranking process.

## Results

The process outlined above and the outcomes of the priority setting partnership following the JLA methodology are summarised in figure 1.

### Gathering uncertainties

Twenty participants, 5 men and 14 women (one did not report gender) answered the pilot questionnaire, which represents a response rate of 57%. The mean age was 38.6 years, ranging from 20 to 64 years, and the respondents were composed of 7 teachers, 10 students and 3 clinicians (table 1). The overall S-CVI was 0.75, meaning that the respondents answered positively for 75% of the uncertainties. The I-CVI regarding relevance was above the recommended 0.78 for 11 uncertainties and just below (0.75–0.7) for 9 of the 27 uncertainties. Regarding understandability, 11 uncertainties were above the recommended I-CVI of 0.78, and 6 were just below (0.75–0.7) (table 2). Uncertainties considered as having both low relevance and understandability were removed; ‘Interprofessional collaboration and changes in practice’ (0.7 respectively 0.5), ‘Disciplinary knowledge and interprofessional collaboration’ (0.5 respectively 0.2), ‘Artificial intelligence and IPE’ (0.7 respectively 0.5) and ‘Simulation and IPE (0.6 respectively 0.4)’. Four uncertainties were rephrased and kept despite a low I-CVI since they were assessed as unclear but relevant by the steering committee; ‘Theoretical models of IPE’ was changed to ‘Pedagogical models for IPE’, ‘Identification/development of robust outcome measures to evaluate IPE’ was changed to ‘Identification and development of reliable outcomes to evaluate IPE’, ‘Timing and scope of IPE’ was changed to ‘When in the education and to what extent should IPE take place’ and ‘The impact of interprofessional collaboration on policies and organisational structures’ was changed to ‘Organisations’ prerequisites for interprofessional collaboration’. Based on suggestions from the participants, ‘Effects of IPE in clinical practice for patients/users/clients/employees’ was divided into two uncertainties: ‘Effects of patients/user/clients’ and ‘Effects for employees’. No further uncertainties were added.

**Table 1:** Age, gender and role of the participants

	Pilot survey n=20	Main survey n=53	Workshop n=14
Age mean (range)	38.6 (20-64)	45.7 (23-67)	n/a
Gender: male/female	5/14 (1 missing)	13/34 (6 missing)	2/12
Role: n		(2 missing)	
Student	10	6	5
Teacher	7	30	4
Clinicians	3	15	5
Workplace: n			
Department of Health and Society, Malmö University,	17	36	9
Skåne University hospital	3	15	5

**Table 2:** The content validity index relevance and understandability of the pilot survey of 27 uncertainties for interprofessional education

Uncertainty	Relevance	Understandability
1 Theoretical models of IPE	0.85	0.5
2 Standardised syllabus/learning outcomes for IPE	0.7	0.8
3 The importance of learning environments for students’ IPE	0.9	0.8
4 Identification/development of reliable measures (outcomes) for evaluating IPE	0.75	0.4
5 Assessment of IPE in different contexts	0.9	0.7
6 Competence of teachers to be able to teach and evaluate IPE	1.0	0.8
7 Preparation of students for IPE	0.8	0.75
8 Methods of interprofessional teaching	0.9	0.75
9 Patients/users/client participation in IPE	0.85	0.7
10 Person-centred care and IPE	0.75	0.7
11 Factors hindering and facilitating interprofessional IPE	0.85	0.9
12 Timing and scope of IPE	0.5	0.65
13 The importance of hierarchical work cultures for IPE	0.7	0.85
14 Implementation of IPL in different contexts	0.85	0.85
15 Long-term follow-ups of the effects of IPE	0.75	0.9
16 Perceptions of and attitudes toward other professions and interprofessional collaboration	0.8	0.85
17 Effects of IPE on the individual student	0.7	0.65
18 Effects of IPE in clinical practice for patients/users/clients/employees	0.9	0.9
19 Effects of IPE learning on patient safety	0.6	0.85
20 Interprofessional collaboration and cost-effectiveness	0.5	0.6
21 The impact of interprofessional collaboration on policies and organisational structures	0.5	0.6
22 Interprofessional collaboration and changes in practice	0.7	0.5
23 Terminology and communication in interprofessional collaboration/education	0.7	0.7
24 Leadership in interprofessional teams	0.65	0.95
25 Disciplinary knowledge and interprofessional collaboration	0.55	0.2
26 Artificial intelligence and IPE	0.7	0.5
27 Simulation and IPE	0.6	0.4

IPE, interprofessional education.

### Interim priority setting

A total of 53 persons (13 men and 34 women, 6 did not report gender) responded to the revised questionnaire. The participants’ mean age was 45.7 years, ranging from 23 to 67 years. Of the participants, 6 were students, 30 were teachers, 15 were clinicians and 2 did not report on their role (table 1). The students were enrolled in the undergraduate nursing programme, and the teachers were from the departments of care science, biomedical science, criminology and social work.

The students ranked ‘Factors that hinder and enable IPE’ as highest, and teachers and clinicians ranked this uncertainty as number 4. In contrast, the teachers ranked ‘Methods of interprofessional teaching’ and ‘Effects of IPE in clinical practice for employees’ as highest. ‘Methods of interprofessional teaching’ was also ranked high as the second most important uncertainty among students and clinicians. However, ‘Effects of IPE in clinical practice for employees’ was not among the highest ranked uncertainties according to students and clinicians. Clinicians ranked ‘Competence for teachers to be able to teach in and evaluate IPE’ and ‘Effects of IPE on patient safety’ as highest, whereas teachers did not rank these uncertainties high at all. Students, however, had the same uncertainties among those considered the second most important. The highest ranked uncertainties among students, teachers and clinicians are shown in table 3.

**Table 3:** Top research uncertainties for interprofessional education from the main survey based on the ranking of students, teachers and clinicians

Uncertainty	Rank	Percentage ranking as very important	Participants
Factors hindering and facilitating interprofessional IPE	1	33.3	Students n=6
Pedagogical models for IPE	2	16.7	
Competence of teachers to be able to teach and evaluate IPE	2	16.7	
Preparation of students for IPE	2	16.7	
Methods of IPE	2	16.7	
Timing and scope of IPE	2	16.7	
The importance of hierarchical work cultures for IPE	2	16.7	
Implementation of IPE in different contexts	2	16.7	
Effects of IPE learning on patient safety	2	16.7	
Methods of interprofessional teaching	1	40.0	Teachers n=30
Effects of interprofessional collaboration in clinical practice for patients/users/clients	1	40.0	
Organisational prerequisites for interprofessional collaboration	2	36.7	
Pedagogical models for IPE	3	33.3	
Effects of IPE learning on patient safety	3	33.3	
Patients/users/client participation in IPE	4	30.0	
Factors that hindering and facilitating IPE	4	30.0	
Perceptions of and attitudes towards other professions and interprofessional collaboration	4	30.0	
The importance of learning environments for students’ IPE	5	26.7	
Identification and development of reliable measures (outcomes) for evaluating IPE	5	26.7	
Competence of teachers to be able to teach and evaluate IPE	1	66.7	Clinicians=15
Effects of IPE learning on patient safety	1	66.7	
Methods of interprofessional teaching	2	60.0	
Person-centred care and IPE	3	53.3	
The importance of learning environments for students’ IPE	4	46.7	
Preparation of students for interprofessional training	4	46.7	
Patients/users/client participation in IPE	4	46.7	
Factors that hindering and facilitating IPE	4	46.7	
Perceptions of and attitudes towards other professions and interprofessional collaboration	4	46.7	

IPE, interprofessional education.

Based on low rankings (less important or not important) in the main survey, three uncertainties were removed before the final workshop: ‘Standardised syllabus/learning outcomes for IPE’, ‘Effects of IPE in clinical practice for employees’ and ‘Interprofessional collaboration and cost-effectiveness’, leaving 21 uncertainties remaining. All ranked uncertainties are presented in online supplemental table.

### Final priority setting

Seventeen persons had registered an interest in taking part in the final workshop. One person withdrew participation and two persons did not turn up, which meant that five students, four teachers and five clinicians took part in the workshop (table 1). In addition, three members from the research team participated. The identified 21 research uncertainties were discussed and ranked during the workshop, as previously described. The participants were divided into three small groups, with five or six members each. The groups were mixed, so that all groups included students, teachers, clinicians and one researcher. After the first ranking steps in the small groups and discussions in the whole group, the participants reached a consensus on the top 10 research priorities (table 4). All participants agreed that the uncertainty ‘Identification/development of reliable measures (outcomes) for evaluating IPE’ was among the highest ranked uncertainty, together with ‘Factors that hinder and facilitate IPE’ and ‘Pedagogical models for IPE’. After a thorough discussion between the participants in the different groups, a consensus was reached regarding the first top three uncertainties. Then the next seven uncertainties were ranked according to each group’s previous discussion and rankings. The uncertainty about ‘Effects of interprofessional collaboration in clinical practice for patients/users/clients’ was ranked as number 10, and the effects on students were added, as the participants considered the effect on students just as important as the other mentioned group of individuals.

**Table 4:** Final top 10 research uncertainties for interprofessional education

Ranking	Uncertainties
1	Identification and development of reliable measures (outcomes) for evaluating IPE
2	Factors hindering and facilitating interprofessional IPE
3	Pedagogical models for IPE
4	Long-term follow-up of the effects of IPE
5	Implementation of IPE in various contexts
6	Competence of teachers to be able to teach and evaluate IPE
7	Organisational prerequisites for interprofessional collaboration
8	Leadership in interprofessional teams
9	Person-centred care and IPE
10	Effects of interprofessional collaboration in clinical practice for patients/users/clients/students

IPE, interprofessional education.

## Discussion

This is to our knowledge the first JLA priority setting partnership that has been performed within the field of IPE. In our endeavour to identify the top 10 priorities for research on IPE, the JLA structure provided a clear process for the involvement of teachers, students and clinicians. This approach proved to be valuable in capturing the perspectives from different groups that will either be conducting research or become the end users. Adapting the JLA process from specific diagnosis-related procedures to the field of IPE proved to work very well. One reason for this could be that the focus was still on healthcare settings.

‘Identification and development of reliable measures (outcomes) for evaluating interprofessional learning’ was agreed by all participants as the top priority, which is unsurprising, as Berger-Estilita *et al*^20^ concluded in a systematic review that few studies report medium and long-term outcomes. Furthermore, Guitar and Connelly^21^ noted a limited number of studies reporting on useful outcome measures and how students can demonstrate achieved learning from IPE activities. Moreover, their review illustrates the vast variety of outcome measures with no consensus on standard outcome measures. Therefore, as the evaluation of outcome measures was ranked as the top priority in our study, we suggest a focus on detailing and developing such measures in collaboration between students, faculty, clinicians and researchers. For example, one such measure was exemplified by Peterson *et al*,^22^ suggesting that further research should address how aggregated IPE initiatives implemented throughout health profession programmes have an impact on self-efficacy for competence in intercollaborative practice.

While the result from our main survey differed slightly between the three groups of participants, we can discern a cluster of uncertainties related to pedagogics as highly rated by all groups. Notwithstanding the existing variation in the rankings, the final priority setting confirmed that ‘Factors hindering and facilitating interprofessional learning’ and ‘Pedagogical models for interprofessional learning’ were rated as number 2 and 3 respectively. This result is probably important to explore following the writings by Mukhalalati and Taylor,^23^ as they stress that healthcare educators are traditionally skilled practitioners yet lack general pedagogical training.

Our result also disclosed that ‘Effects of interprofessional collaboration in clinical practice for patients/users/clients/students’ was the lowest ranked uncertainty. While effects for students are rather well studied and described as an increased awareness of others’ professional competence,^24^ increased collaborative skills and teamwork abilities,^25^ and increased self-efficacy in competence in interprofessional collaborative practice,^26^ we argue that too few studies explore the effects in clinical practice of interprofessional collaboration from the patient perspective. Thereby, we recommend, first, exploring the effects of IPE, including the voice of patients and their families, and second, inviting patients as active partners when setting priorities for research agendas, thus directly affecting patient care given by interprofessional teams by employing the JLA structure. This is particularly important, as a recent study^27^ discloses that patients do not feel included or are invited to participate in their care, resulting in the loss of information that is vital for continuous care planning. This is also acknowledged in a study by Jensen *et al*,^28^ which stresses the significant role of the patient to promote IPE as a patient-centred practice by contributing with key information to healthcare providers and should therefore be involved in decision-making.

Finally, we found it somewhat alarming that ‘Interprofessional collaboration and learning and its impact on cost effectiveness’, although being one of our primary questions in the pilot survey, only showed a CVI of 0.5 for relevance and 0.6 for understandability and was thereby excluded at an early stage. We base this concern on previous research reporting that interprofessional collaboration is one way to reach cost-effectiveness across healthcare systems.^1 29^ Furthermore, a recent report by the International Council of Nurses^30^ emphasises the importance of multidisciplinary teams and shared leadership, with all team members contributing to patient care, as delays to healthcare may lead to increased costs. Therefore, our reflection and lesson learnt is that financial issues cannot, and should not, be avoided in future research, as most healthcare systems globally are under pressure due to a growing older population, increased healthcare costs and challenges with the attrition and retention of healthcare professionals.

One limitation of this priority-setting partnership may be the low response rate to the main survey. In particular, the limited participation of students may have influenced the ranking of priorities. However, student rankings were reported separately and, in line with the JLA process, were given equal weight to those of teachers and clinicians. Furthermore, the JLA guidance does not recommend a specific sample size, emphasising quality over quantity.^9^ The limited number of students was partly compensated for during the workshop by dividing participants into mixed small groups in which students, teachers, clinicians and researchers were evenly represented. The lack of patients in the priority setting partnership could be another limitation, as patients are important team members, and their engagement in care is essential for optimal health outcomes.^31^ However, this project was not focused on the outcome of IPE but rather more on educational aspects that may have been difficult for patients to prioritise which was the reason for excluding patients during the whole process. Nevertheless, we suggest that in line with findings from the study by Hemle Jerntorp^27^ that patients, if invited to the current study, have highlighted the importance of studying how interprofessional teams can be more conscious of the importance of including patients as active and equal partners in their care. Furthermore, the rankings of research uncertainties might have been different if this JLA priority setting partnership was exclusively about the students’ perspective. In that case, more attention would probably have been paid to the competence of the teachers or student preparation for IPE. Nevertheless, the current priority setting partnership process provided an actual list of research priorities to address in future research aimed at IPE. In addition, the top 10 list of research priorities may to some extent reflect a Swedish context and therefore may not be entirely applicable to other countries. The purpose of the top 10 ranked research priorities is to highlight important aspects of IPE for future research rather than to formulate specific research questions. These priorities typically represent broad areas that need to be translated into concrete research questions for individual studies. The optimal outcome of a JLA priority-setting partnership is that one or more of the identified priorities are developed into research studies that have a meaningful impact on those affected.

## Conclusion

The top 10 research priorities for IPE represent consensus areas from students, teachers and clinicians that can guide future research within this area. These research priorities can further justify and inform the strategic allocation of research funding. Moreover, the extended use of the JLA process including students and teachers was deemed feasible.

## Supplementary material

10.1136/bmjopen-2025-113426 online supplemental file 1

## Data Availability

Data are available upon reasonable request.
