# Supplementary material for: Research priorities in the field of interprofessional healthcare education: a James Lind Alliance Priority Setting Partnership
Source: BMJ Open. 2026 May 14;16(5):e113426. doi: 10.1136/bmjopen-2025-113426 (PMC13182379; doi:10.1136/bmjopen-2025-113426)
Supplement: online supplemental file 1 [file bmjopen-16-5-s001.docx]

Supplemental table: All ranked uncertainties included in the main survey, n (%)

| **Uncertainty** | **Very important** | **Important** | **Less important** | **Not important** | **Missing=n** |
| --- | --- | --- | --- | --- | --- |
| Pedagogical models for IPE | 17 (32.1) | 33 (62.3) | 3 (5.7) | - |  |
| Standardised syllabus/intended learning outcomes for IPE** | 5 (9.4) | 26 (49.1) | 21 (39.6) | 1 (1.9) |  |
| The importance of learning environments for students' IPE | 15 (28.3) | 31 (58.5) | 6 (11.3) | 1 (1.9) |  |
| Identification and development of reliable measures (outcomes) for evaluating IPE | 15 (28.3) | 26 (49.1) | 11 (20.8) | 1 (1.9) |  |
| Assessment of IPE in different contexts | 12 (22.6) | 31 (58.5) | 10 (18.9) | - |  |
| Competence of teachers to be able to teach and evaluate IPE | 19 (35.8) | 28 (52.8) | 6 (11.3) | - |  |
| Preparation of students for IPE | 14 (26.4) | 29 (54.7) | 7 (13.2) | 2 (3.8) | 1 |
| Methods of interprofessional teaching | 23 (43.4) | 25 (47.2) | 5 (9.4) | - |  |
| Patients'/Users'/Clients' participation in IPE | 17 (32.1) | 28 (52.8) | 8 (15.1) | - |  |
| Person-centred care and IPE | 16 (30.2) | 30 (56.69 | 6 (11.3) | - | 1 |
| Factors that hinder and facilitating IPE | 18 (34.0) | 28 (52.8) | 6 (11.3) | - | 1 |
| When in the education and to what extent should IPE take place | 10 (18.9) | 30 (56.6) | 12 (22.6) | 1 (1.9) |  |
| The importance of hierarchical work cultures for IPE | 15 (28.3) | 25 (47.2) | 11 (20.8) | 1 (1.9) | 1 |
| Implementation of IPE in different context | 13 (24.5) | 32 (60.4) | 7 (13.2) | - | 1 |
| Long-term follow-up of the effects of IPE | 10(18.9) | 28 (52.8) | 13 (24.5) | - | 2 |
| Perceptions of and attitudes towards other professions and interprofessional collaboration. | 16 (30.2) | 23 (43.4) | 12 (22.6) | 2 (3.8) |  |
| Effects of IPE on the individual student | 11 (20.8) | 24 (45.3) | 14 (26.4) | 3 (5.7) | 1 |
| Effects of interprofessional collaboration in clinical practice for patients/users/clients | 18 (34.0) | 28 (52.8) | 5 (9.4) | 1 (1.9) | 1 |
| Effects of interprofessional collaboration for employees** | 11 (20.8) | 18 (34.0) | 19 (35.8) | 3 (5.7) | 2 |
| Effects of IPE on patient safety | 22 (41.5) | 23 (43.4) | 4 (7.5) | 2 (3.8) | 2 |
| IPE and cost-effectiveness** | 8 (15.1) | 20 (37.7) | 21 (39.6) | 2 (3.8) | 2 |
| Organisations' prerequisites for interprofessional collaboration | 17 (32.1) | 21 (39.6) | 13 (24.5) | - | 2 |
| Terminology and communication in IPE | 10 (18.9) | 25 (47.2) | 16 (30.2) | 1 (1.9) | 1 |
| Leadership in interprofessional teams | 10 (18.9) | 29 (54.7) | 11 (20.8) | - | 3 |

** Items removed in the final priority setting workshop
