# Supplementary material for: Research priorities in the field of interprofessional healthcare education: a James Lind Alliance Priority Setting Partnership
Source: BMJ Open. 2026 May 14;16(5):e113426. doi: 10.1136/bmjopen-2025-113426 (PMC13182379; doi:10.1136/bmjopen-2025-113426)
Supplement: Supplementary file 2 [file bmjopen-2025-113426.reviewer_comments.pdf]

PEER REVIEW HISTORY

BMJ Open publishes all reviews undertaken for accepted manuscripts. Reviewers are asked to complete a checklist review form and are provided with free text boxes to elaborate on their assessment. These free text comments are reproduced below.

ARTICLE DETAILS

Title (Provisional)

Research priorities in the field of interprofessional healthcare education: A James Lind Alliance Priority Setting Partnership

Authors

Kumlien, Christine; Ask, Elliotte; Axelsson, Malin; Gudmundsson, Petri; Hugosson, Olivia; Jakobsson, Jenny; Stollenwerk, Maria; Carlson, Elisabeth

---

VERSION 1 - REVIEW

**Reviewer**	**1**
**Name**	**Gravensteen, Ida Kathrine**
**Affiliation**	**Universitetet i Oslo**
**Date**	**13-Dec-2025**
**COI**	**None**

General comments

This manuscript addresses an important and timely topic: The need to identify relevant research priorities in interprofessional healthcare education. The use of a James Lind Alliance Priority Setting Partnership (JLA PSP) in an educational context is novel and potentially valuable, particularly given the increasing emphasis on stakeholder involvement in research agenda-setting.

The paper is generally well written, the language is clear, and the limitations are acknowledged by the authors. The topic will likely be of interest to BMJ Open readers, especially those involved in healthcare education and curriculum development.

However, the manuscript would benefit from a clearer and more transparent description of the study design and methodological decisions, particularly to allow readers to assess validity and replicability.

Major comments

1. Clarity and transparency of study design

The manuscript includes a flowchart outlining the JLA process; however, the figure is too schematic to convey how key methodological decisions were made at each stage (e.g. selection, modification, and exclusion of uncertainties). Additional details are needed to allow assessment of methodological rigor and replicability.

2. Selection and formulation of research uncertainties

It is unclear how the steering committee was constituted and how decisions were made regarding:

o which uncertainties were initially included

o how guideline recommendations were translated into survey items

o how uncertainties were revised, merged, or removed following the pilot survey

More detail is needed to understand how subjective judgments were managed and to assess the risk of confirmation bias. Without this information, it is difficult to evaluate the replicability of the study.

3. Pilot survey methodology and its impact on the main survey

While conducting a pilot survey is a strength, the reporting of the pilot methodology is limited. The authors should clarify:

o how participants were recruited

o how many were invited

o how pilot findings specifically informed changes to the main survey

4. Sampling strategy and representativeness

The manuscript reports sample sizes but provides scarce information about the target population, recruitment strategy, or response rate. This makes it difficult to assess representativeness and potential selection bias. This information is essential, particularly for a priority-setting exercise intended to guide future research agendas.

5. Rationale for choosing the JLA methodology

Given that BMJ Open readers may have limited familiarity with educational research methods, the authors should more explicitly justify why the JLA approach was chosen over alternative priority-setting or consensus methods (e.g. Delphi processes), and discuss its suitability for this educational context.

6. Lack of patient involvement

The absence of patient or public involvement is acknowledged as a limitation. However, further reflection would be valuable on why patients were excluded at all stages, and how their absence may have influenced the identified priorities.

Minor comments

• Several editorial issues should be addressed, including placeholder text (e.g. “XX University”) and minor typographical errors. Thorough proofreading is recommended.

• An appendix listing the full wording of all research uncertainties would improve transparency and allow readers to better interpret the prioritization process.

Overall assessment

In summary, this study addresses a relevant and important topic and applies a novel methodological approach. However, the manuscript requires clearer and more detailed reporting of the methodological process to allow readers to assess external validity and replicability. With revisions addressing the points above, the paper has the potential to make a valuable contribution to the field of interprofessional education research.

Reviewer **2**

Name **Coller , Ryan J.**

Affiliation **University of Wisconsin-Madison School of Medicine and Public Health**

Date **02-Mar-2026**

COI **None**

This manuscript provides a nicely written summary of a consensus method (James Lind Alliance Priority Setting Partnership) to establish a set of 10 research priorities for interprofessional healthcare education.

-Including participants from different backgrounds / disciplines is a strength; however, it'd be helpful to explain in more detail the range of disciplines included (and those missing)

-Explaining how participants were selected will help understand more about the "expertise of the experts"

-Lack of patient inclusion is a limitation the authors recognize.

-The methods are otherwise generally well described. The phrase "interprofessional education" is pretty broad, and clearly defining what it means (and what it doesn't) would help readers. What are the contexts for delivering interprofessional education? If one is a medical student or nursing student (or a physician or a nurse), when / where will they experience IPE?

-The articulated priorities that result from the study remain pretty general. It feels like many of them (e.g., factors hindering and facilitating IPE) could have their own subsequent priority setting activities given how wide and sweeping they are. It'd be helpful for the authors to discuss how users should actually approach this ranked list, since it still encompasses (a potentially overwhelming) amount of topics / content (arguably almost

-a table describing summary statistics of the participants would help readers understand who the authors reached in conducting the study.

Reviewer 3

Name Schwill, Simon

Affiliation Heidelberg University, General Practice and Health Services Research

Date 18-Mar-2026

COI None

Dear Colleagues, thank you very much for the opportunity to review this manuscript. I was wondering

- what makes your approach relevant to international readers and other faculties, is this a best practice paper? is this a "how to" paper?, please point this out and be clear aboiut it

- this is also linked to your research question: if you see it as the begining of a curricular change in regard to KERN et al, this is a analysis / needs assessment, and next steps would be how you would like to implement any measures to affect your areass of interest

- if this is one of the first testings of JLP-Seeting please point out, what makes your study unique?

- this also affects the discussion / conclusion

m&M

please add ethics, please add more details on the experts - is it a stakeholder group, will the ideads be implemented into the curirculum? --> elaborate in the discussion

discussion: how does your finding link to which curricula: nursing? medicine? physiotherapy? "over-all basis health professional skill"? It would benefit from explanations in the introduction on which courses are offered and your institution and how your research will affect this

all together I think this is a promising approach and sound paper, but clarifications and major revisions are needed to answer my querries, (so what? who cares?)

kind regards

VERSION 1 - AUTHOR RESPONSE

Reviewer: 1
Ms. Ida Kathrine Gravensteen, Universitetet i Oslo

General comments

This manuscript addresses an important and timely topic: The need to identify relevant research priorities in interprofessional healthcare education. The use of a James Lind Alliance Priority Setting Partnership (JLA PSP) in an educational context is novel and potentially valuable, particularly given the increasing emphasis on stakeholder involvement in research agenda-setting.

The paper is generally well written, the language is clear, and the limitations are acknowledged by the authors. The topic will likely be of interest to BMJ Open readers, especially those involved in healthcare education and curriculum development. However, the manuscript would benefit from a clearer and more transparent description of the study design and methodological decisions, particularly to allow readers to assess validity and replicability.

Major comments

1. Clarity and transparency of study design

The manuscript includes a flowchart outlining the JLA process; however, the figure is too schematic to convey how key methodological decisions were made at each stage (e.g. selection, modification, and exclusion of uncertainties). Additional details are needed to allow assessment of methodological rigor and replicability.

Thank you, we can now see that this is somewhat unclear and have tried to clarify our key methodological decisions both in the text and in the figure as described more in detail below.

2. Selection and formulation of research uncertainties

- It is unclear how the steering committee was constituted and how decisions were made regarding:
  which uncertainties were initially included?
- how guideline recommendations were translated into survey items?

The constitution of the steering committee is explained more in detail in the text (page 6, line 147 and 160) and in figure 1. Further, on how decisions were made regarding the initial included uncertainties (page 7, line 172).

- how uncertainties were revised, merged, or removed following the pilot survey?

More detail is needed to understand how subjective judgments were managed and to assess the risk of confirmation bias. Without this information, it is difficult to evaluate the replicability of the study.

We have described the process how the selection of uncertainties was performed more in detail in the result section (page 9, line 255). However, according to the JLA guidebook, research uncertainties are typically informed by service users (students and teachers) with experience in the area of interest (IPE), healthcare professionals, and existing guidelines and systematic reviews. This means that subjective judgements from all involved partners exist as

an accepted part of the process. This is now explained in the methods for clarity (page 7, line 172).

3. Pilot survey methodology and its impact on the main survey
While conducting a pilot survey is a strength, the reporting of the pilot methodology is limited. The authors should clarify:

- how participants were recruited?

This information is now added at page 7, line 185.

- how many were invited?

We see that this information unfortunately is missing and 35 persons were invited to answer the pilot questionnaire. This is now added in the text on page 7, line 185.

- how pilot findings specifically informed changes to the main survey?

Please see above. This process is clarified in the result section, page 9, line 258.

4. Sampling strategy and representativeness
The manuscript reports sample sizes but provides scarce information about the target population, recruitment strategy, or response rate. This makes it difficult to assess representativeness and potential selection bias. This information is essential, particularly for a priority-setting exercise intended to guide future research agendas.

We agree that this information would make it easier to replicate the study and assess the representativeness of the participants. However, in this case, information about participation together with a link to the survey was posted at the faculty internal website for teachers and at the learning platform CANVAS for students in the biomedical scientist program (semester 4) and nursing program (semester 4 and 6). Clinicians taking part in an ongoing preceptor preparation course got information and weblink through the same CANVAS platform as the students. For this recruitment strategy we estimated to reach approximately 600 possible participants. Therefore, is a correct response rate impossible to calculate. We have tried to describe this more in detail in the method (Page 8, line 203) and in the discussion (Page 13, line 369) as a limitation. However, according to the JLA guidance there is no recommended maximum or minimum of responses, and the extent will depend on the topic, audience and resource constraints. They further state that the priority setting partnership should aiming for quality not quantity.

5. Rationale for choosing the JLA methodology
Given that BMJ Open readers may have limited familiarity with educational research methods, the authors should more explicitly justify why the JLA approach was chosen over alternative priority-setting or consensus methods (e.g. Delphi processes), and discuss its suitability for this educational context.

We have now argued for chosen the JLA methodology in the beginning of the method section (page 6 , line 152) and in the discussion (page 11, line 321).

6. Lack of patient involvement
The absence of patient or public involvement is acknowledged as a limitation. However, further reflection would be valuable on why patients were excluded at all stages, and how their absence may have influenced the identified priorities.

We agree that this is a shortcoming with this priority setting project and we have written additional text about why they were excluded and how their absence may have influenced the identified priorities (Page 13, line 382).

Minor comments

• Several editorial issues should be addressed, including placeholder text (e.g. “XX University”) and minor typographical errors. Thorough proofreading is recommended.

A thorough proofreading has been performed, and the editorial issues are removed.

• An appendix listing the full wording of all research uncertainties would improve transparency and allow readers to better interpret the prioritization process.

We have added a supplemental table with the full wording of all research priorities from the main survey and the final priority setting workshop.

Overall assessment

In summary, this study addresses a relevant and important topic and applies a novel methodological approach. However, the manuscript requires clearer and more detailed reporting of the methodological process to allow readers to assess external validity and replicability. With revisions addressing the points above, the paper has the potential to make a valuable contribution to the field of interprofessional education research.

Thank you very much for your valuable comments and suggestions that we believe substantially have improved the manuscript.

Reviewer: 2
Ryan J. Coller, University of Wisconsin-Madison School of Medicine and Public Health

Comments to the Author:

This manuscript provides a nicely written summary of a consensus method (James Lind Alliance Priority Setting Partnership) to establish a set of 10 research priorities for interprofessional healthcare education.
-Including participants from different backgrounds / disciplines is a strength; however, it'd be helpful to explain in more detail the range of disciplines included (and those missing)

We agree that this could have strengthen the representativity of the sample. However, those who answer the survey could only chose between the response options; student, teacher or clinicians so we are not able to give any further information about the disciplines.

-Explaining how participants were selected will help understand more about the "expertise of the experts"

We have now added further information about the selection and recruitment at page 7, line 185 and page 8, line 203.

-Lack of patient inclusion is a limitation the authors recognize.

Yes, we agree and this is further developed in the section of limitations. (Page 13, line 378)

-The methods are otherwise generally well described. The phrase "interprofessional education" is pretty broad, and clearly defining what it means (and what it doesn't) would help readers.

We had defined IPE in the background based on the WHO definition, however, to make it more clear we have slightly changed the definition and refer to WHO (2010).

What are the contexts for delivering interprofessional education? If one is a medical student or nursing student (or a physician or a nurse), when / where will they experience IPE?

Thank you and this is a valid question. IPE as described in the literature may take many forms stretching from brief interventions of 1-2 hours to prolonged interprofessional learning activities during a full semester. Also, the interprofessional healthcare teams of students participating in IPE activities can be composed of any healthcare profession if at least two different professions are represented. IPE activities can take place in clinical practice as well as in campus-based activities. Thus, IPE provides a rich and complex structure for learning and as the IPE activities in itself are not in focus in the current study we have chosen not to elaborate further.

-The articulated priorities that result from the study remain pretty general. It feels like many of them (e.g., factors hindering and facilitating IPE) could have their own subsequent priority setting activities given how wide and sweeping they are. It'd be helpful for the authors to discuss how users should actually approach this ranked list, since it still encompasses (a potentially overwhelming) amount of topics / content (arguably almost

The purpose of the top 10 ranked research priorities is to highlight important areas for future research rather than to formulate specific research questions. These priorities typically represent broad areas that need to be translated into concrete research questions for individual studies. The optimal outcome of a JLA priority-setting partnership is that one or more of the identified priorities are developed into research studies that have a meaningful impact on those affected. We have now developed the text regarded this in the discussion (P 13, line 391).

-a table describing summary statistics of the participants would help readers understand who the authors reached in conducting the study.

Thank you for the suggestion and we have added a new table 1 with the available information from the surveys and the workshop.

Reviewer: 3
Dr. Simon Schwill, Heidelberg University

Comments to the Author:
Dear Colleagues, thank you very much for the opportunity to review this manuscript. I was wondering
- what makes your approach relevant to international readers and other faculties, is this a best practice paper? is this a "how to" paper?, please point this out and be clear about it

The purpose of the top 10 ranked research priorities is to highlight important areas for future research rather than to formulate specific research questions. These priorities typically represent broad areas that need to be translated into concrete research questions for

individual studies. The optimal outcome of a JLA priority-setting partnership is that one or more of the identified priorities are developed into research studies that have a meaningful impact on those affected. We have now developed the text regarded this in the discussion (P 13, line 391).

- this is also linked to your research question: if you see it as the beginning of a curricular change in regard to KERN et al, this is a analysis / needs assessment, and next steps would be how you would like to implement any measures to affect your areas of interest

Please see the answer above. The aim was not to change any curriculum or use the six-step approach to curriculum development framework by Kern. In fact, the uncertainty about "Standardized syllabus/learning outcomes for IPE" was ranked low in the main survey and removed before the final priority setting workshop.

- if this is one of the first testing of JLP-Setting please point out, what makes your study unique?

Thank you for reminding us since this is to our knowledge the first JLA priority setting partnership that have been performed within the field of interprofessional education from the view of students, teachers and clinicians. This is now added in the beginning of the discussion (page 11, line 316).

- this also affects the discussion / conclusion

As stated above this is now added in the beginning the discussion.

Methods:

-please add ethics

According to author guidelines for BMJ open is the section about ethics located after the references.

-please add more details on the experts - is it a stakeholder group, will the ideas be implemented into the curriculum? --> elaborate in the discussion

We have summarised the available details about the participants in each step of the process in a new table 1. Please note that the aim was not to develop a new curriculum.

Discussion:

-how does your finding link to which curricula: nursing? medicine? physiotherapy? "over-all basis health professional skill"? It would benefit from explanations in the introduction on which courses are offered and your institution and how your research will affect this

As mentioned above the aim was not to develop a new curriculum. However, the students and teachers who participated in the priority setting partnership came mainly from the undergraduate biomedical scientist and nursing programs.

all together I think this is a promising approach and sound paper, but clarifications and major revisions are needed to answer my queries, (so what? who cares?)

VERSION 2 - REVIEW

**Reviewer**	**2**
**Name**	**Coller , Ryan J.**
**Affiliation**	**University of Wisconsin-Madison School of Medicine and Public Health**
**Date**	**24-Apr-2026**
**COI**	

The authors have responded to most reviewer comments and the manuscript is improved.
